# Spotted fever rickettsioses causing myocarditis and ARDS: a case from Sri Lanka

**DOI:** 10.1186/s12879-018-3631-6

**Published:** 2018-12-29

**Authors:** H. M. L. Y. Herath, J. M. H. D. Jayasundara, S. D. N. Senadhira, S. A. M. Kularatne, W. K. S. Kularatne

**Affiliations:** 1 Department of General Medicine, General Hospital (Teaching), Kandy, Sri Lanka; 2 Department of Medicine, Faculty of Medicine, University of Peradeniya, Kandy, Sri Lanka

**Keywords:** Spotted fever, Rickettsia conorii, Myocarditis, Acute respiratory distress syndrome, ARDS

## Abstract

**Background:**

Spotted fever group of rickettsial infections are emerging in Sri Lanka. We describe a patient with rapidly progressing ARDS and myocarditis secondary to spotted fever caused by *Rickettsia conorii*. ARDS and myocarditis are rare complications of *Rickettsia conorii* infections and only a few cases are reported to date.

**Case presentation:**

A 53 years old manual worker presented with fever for 5 days and a skin rash. He was in circulatory failure on admission and developed severe hypoxaemia with gross changes in chest radiograph by next day requiring assisted ventilation. He had myocarditis causing left ventricular failure and acute respiratory distress syndrome. He was confirmed to have spotted fever rickettsial infection with rising titre of indirect immunofluorescence antibodies to *Ricketssia conorii* and made a complete recovery with appropriate antibiotic therapy and supportive care.

**Conclusion:**

Rickettsial infections can present with diverse manifestations. Even the patients with severe organ involvements such as myocarditis and ARDS can be completely cured if timely identified and treated.

## Background

Emergence of spotted fever group of rickettsial infections in the hilly central province of Sri Lanka was first observed in early nineties [1]. *Rickettsia conorii,* the organism known to cause Mediterranean spotted fever (MSF) is the most prevalent organism causing spotted fever in Sri Lanka. Few serologically confirmed cases of *Rickettsia honei* and *Rickettsia japonica* has also been reported [1, 2].

Usual presentation of spotted fever is with a prodrome of high grade fever, headache myalgia, arthralgia and anorexia. Less common manifestations include frank arthritis, cough, abdominal pain, conjunctival injection and diarrhea [1]. Various neurological manifestations including confusion, hallucinations, tinnitus, hearing impairment and rarely coma are also seen [3]. The characteristic skin rash is present only in about 40% of patients. Typically, the rash is maculopapular with predominant involvement of limbs including palms and soles. In severe cases fern leaf type skin necrosis can occur. The typical eschar is rare to be found and often the patients are unaware of of tick bites [1, 4]. On rare occasions patients present with fever and multiple organ dysfunction making it difficult for the clinician to find the exact diagnosis since many tropical diseases can cause a similar picture. Indirect immunofluorescent antibody assay (IFA) is the reference serology method for the diagnosis. It is available in only a few laboratories in Sri Lanka. Limited availability of IFA has led to underreporting of the cases with rickettsial infections. MSF is usually a mild disease with a mortality rate around 2.5%. Elderly patients are prone to get more complications [5]. Mortality data regarding Sri Lankan patients are not available, except few fatal case reports.

The following case report highlights myocarditis and acute respiratory distress syndrome (ARDS) as complications in a severely ill patient with spotted fever group of rickettsioses where timely diagnosis and intervention saved the life.

## Case presentation

A 53-year-old male was transferred from Peripheral Hospital Hatharaliyadda (PHH) to Teaching Hospital, Kandy (THK) in a state of circulatory failure for specialized care. He was a previously well ‘tree cutter’ working close to his residence situated in a hilly terrain in the Northern slope of central hills of Sri Lanka where rich lust green vegetations and tropical trees are in abundance. His routine was to cut trees in the tea estates in the area and to carry the logs to the closest motorable road.

**Fig. 1:**
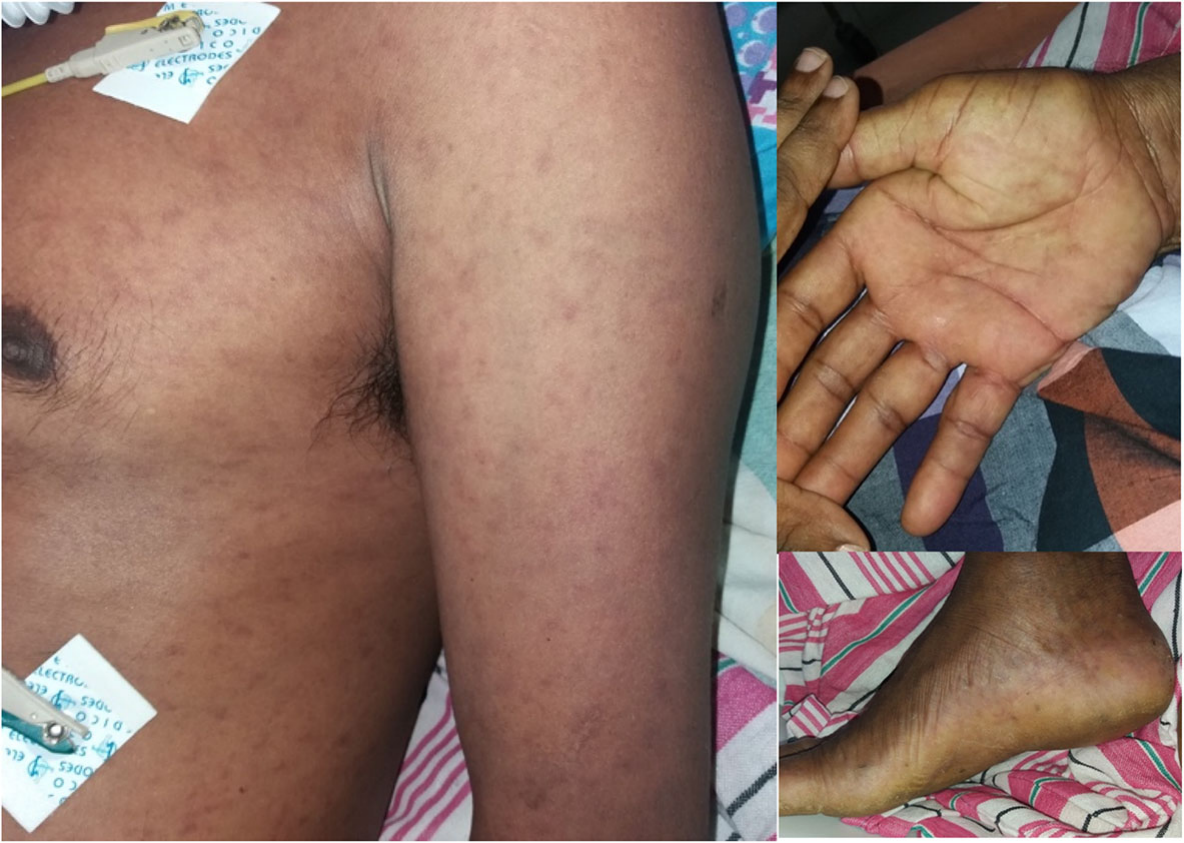
Skin rash. Skin rash of the patient at the day of presentation. It was erythematous macular rash involving palms and soles with mild pedal edema

He developed fever with myalgia and headache 5 days prior to the admission to PHH. On the 4th day of fever he had noticed a rash over his body. As his condition deteriorated on the 6th day of the illness, he was transferred to THK. On admission, he was febrile and recorded temperature was 102 °F. He had a generalized discrete erythematous macular rash in most areas of the body including palms and soles. Also he had swelling of both ankle joints. He denied any tick bite prior to illness. There was no eschar found. He had neither lymphadenopathy nor splenomegaly. But the liver was palpable 2 cm below costal margin. Lungs were clear to auscultation. (Fig. 1) He had a thready pulse of 100/min with a blood pressure of 80/50 mmHg.

He was initially resuscitated with intravenous normal saline and managed as septic shock. The presence of skin rash prompted to consider a spotted fever rickettsial infection. Therefore, he was started on intravenous ceftriaxone and oral doxycycline. Since his blood pressure did not improve with fluid resuscitation a central line was placed and intravenous norepinephrine infusion at a rate of 0.4μg/kg/min was commenced along with septic dose of intravenous hydrocortisone 50 mg/6hourly. His plasma random glucose was normal. Initial electrocardiogram did not show ST/T wave abnormalities and Chest radiograph was normal. His serial investigations during the hospital stay are shown in Table 1.

**Table 1:** Serial Investigations

Day of illness	1	2	3	4	5	6	7	8	9
WBC × 10^9^	11.7	20	29.2	12.12	7.58	8.4	8.75	6.79	8.35
Neutrophil%	70	91	89	90	82	79	82	84	78
Lymphocyte%	17	2.7	5	6	13	15	12	10	15
Hemoglobin g/l	13.6	15.2	13.6	11.8	12.7	11.2	9.7	9.2	9.2
Platelets × 10^9^	215	272	358	270	292	232	180	162	147
INR	0.98						1.05		
APTT (s)	28						30		
ALT U/l	53	124	68	53.7	40	60	84	75	73
AST U/l	133	292	107	71	51	151	138	85	90
Creatinine µmol/l	95	128	118	101	81	85	59	62	54
Total Bilirubin µmol/l	15.7		30	23					
Direct Bilirubin µmol/l	11.2		23	15					
C Reactive Protein mg/dl	139				92				55
Procalcitonin ng/ml		5.78							0.61
pO2 mmHg		75	114	136		134		142	
pCO2 mmHg		20	25	38		42		40	
pO2/FiO2		125	228	218		252		249	

**Fig. 2:**
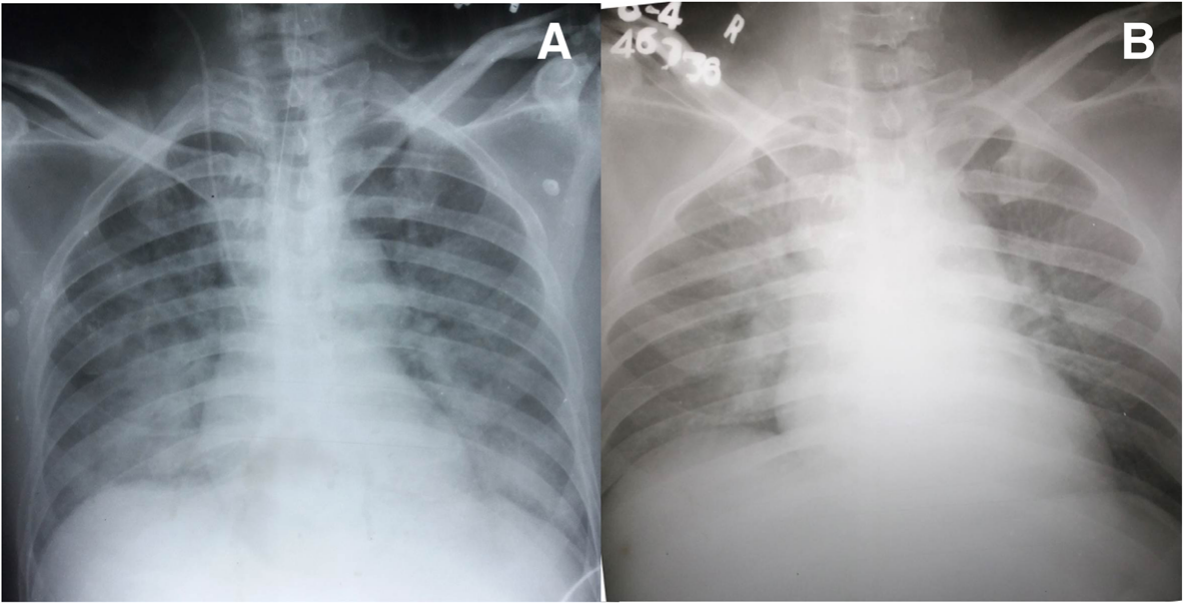
Chest Radiographs. A - Chest radiograph on day 2 of the illness with bilateral diffuse alveolar shadows. B - Chest radiograph at the time of discharge with clearing of alveolar shadows

With inotropic support, antibiotics and maintenance fluids he remained stable for the next 36 h. His blood cultures, urine cultures and retroviral studies were negative. During the latter part of the second day of admission to THK he developed progressively worsening shortness of breath with hypoxemia and hypotension. Blood gas analysis showed type 1 respiratory failure with pO_2_/FiO_2_ ratio of 135.8. Repeat chest radiograph showed bilateral alveolar and interstitial shadowing of both upper and mid zones. ECG revealed sinus tachycardia with no significant ST/T wave changes. 2D Echo cardiogram showed a ventricular ejection fraction of 40–45% with global hypokinesia of myocardium suggestive of myocarditis. Troponin-I titre was positive at 4.9 ng/ml (Normal < 0.12 ng/ml) and NTproBNP (N-terminal pro b-type natriuretic peptide) value was elevated at 11954 pg/ml. At this juncture, elective intubation was done and the patient was transferred to the Intensive Care Unit for assisted ventilation. (Fig. 2).

The management team identified some issues with regard to diagnosis and choosing the appropriate treatment for the patient. Echocardiographic findings, elevated troponin titre and elevated BNP were consistent with myocarditis leading to heart failure causing pulmonary oedema and hypoxemia. The possibility of ARDS was considered based on clinical, blood gas and radiological evidence. Spotted fever was considered to be the most likely diagnosis because of the presence of the typical rash. Other differential diagnoses included leptospirosis with myocarditis and pneumonitis. But the rash and lack of liver and renal involvement was not in favor of leptospirosis. Streptococcal and Staphylococcal toxic shock syndromes and meningococcal sepsis were also taken into consideration, but inability to fulfill diagnostic criteria and persistently negative cultures were against them. Hemophagocytic lymphohistiocytosis (HLH) is also known to complicate many zoonoses including spotter fever infections [6, 7]. However, absence of cytopenias and the splenomegaly made it less likely and further screening tests for HLH were not performed.

Considering the poor response to previous antibiotics, intravenous chloramphenicol 500 mg 6 hourly was added to the treatment regimen as authors personally had seen good response particularly in patients with severe disease. He required 3 inotropes in increasing doses, including norepinephrine 0.5μg/kg/min, Dobutamine 10μg/kg/min and Dopamine 15μg/kg min, to maintain the blood pressure for the next 24 h. In view of myocarditis, IV hydrocortisone dose was increased to 200 mg/6hourly. Within 12 h from starting chloramphenicol and increasing the dose of steroids, his clinical parameters started to improve. By next day tailing off of the inotropic support was possible. On the 3rd day in the Intensive Care Unit, he was extubated and on the 4th day he was transferred back to the High Dependency Unit of the medical ward. The skin rash started to fade leaving few necrotic areas over the hands. All three antibiotics were stopped after the completion of 7 days. He did not develop any treatment related complications. He was recovered enough to be discharged from the hospital on the 10th day of admission.

In two weeks’ time, he was reviewed in the out-patient clinic and found to be completely asymptomatic. For the confirmation of diagnosis, 1st acute blood sample was tested for *R. conorii* indirect immunofluorescence antibodies (IFA) at the reference laboratory of University of Peradeniya which showed moderately high positive titre (1/256). Ten days later, 2nd blood sample was tested with IFA which showed rising titre (1/1024) confirming the diagnosis. Further species identification was not possible due to unavailability of facilities. Unfortunately patient did not turn up for the follow up echocardiogram.

## Discussion and conclusions

We presented a middle aged man, a tree cutter in profession falling ill with fever, then collapsed on 6th day of illness due to myocarditis and became hypoxic due to ARDS. Timely diagnosis of spotted fever and initiation of appropriate treatment saved his life. Even though he denied a tick bite, it was likely that he had an unnoticed tick bite as his occupation carried a high risk of exposure. This case demonstrates a rather rare presentation of spotted fever rickettsial infection where patient deteriorated within short time leading to shock and ARDS. The development of myocarditis was rapid and severe enough to cause low left ventricular ejection fraction and hypotension.

Patients with myocarditis and ARDS are described in the literature in other types of rickettsial infections particularly with scrub typhus. Myocarditis has been observed as an autopsy finding in fatal cases of Rocky Mountain spotted fever. Other much rarer forms of tick borne rickettsial infections like *Sibirica mongolitimonae* infections are also known to cause clinically significant myopericarditis [8]. *R. conorii* related cardiac involvement is extremely rare and only about 6 cases are described in the literature [9]. Severe forms of spotted fever rickettsioses is also known to be caused by some subspecies such as *Rickettsia conorii subsp. israelensis* [10]. Unfortunately molecular diagnostic methods for identifications of subspecies is not yet available in Sri Lanka.

Myocarditis is caused mainly by viruses and also by leptospira spirochetes and toxins. In Sri Lanka myocarditis is an occasional complication in dengue infection and leptospirosis [11]. It is not a diagnosis entertained in rickettsial infections despite its high prevalence. In myocarditis, patients usually develop undue tiredness, chest discomfort and dyspnoea which may progress to cardiogenic shock or development of arrhythmias. Most often it is diagnosed clinically with ECG abnormalities such as T wave inversions, bundle branch blocks and presence or rhythm abnormalities. Echocardiography and elevation of cardiac biomarkers can be used to diagnose myocarditis but these become evident mostly in severe cases. In the background of sepsis, transient cardiac dysfunction can occur due to sepsis induced cardiomyopathy. Differentiation between the two diagnoses can only be achieved by endomyocardial biopsy. However, in acutely ill patients it is not justifiable do biopsy as it does not alter the management. Further, in the available literature suggests that rickettsial infections related cardiac dysfunction is more likely to be due to myocarditis [8, 9, 12]. Newer methods including cardiac MRI and segmented inversion recovery gradient-echocardiography pulse sequences have a better sensitivity in diagnosing acute myocarditis [13, 14]. Our patient had compatible symptoms and signs with global hypokinesia in 2D Echocardiogram and elevated troponin and NTproBNP values to suggest the presence of myocarditis.

Differentiation between severe pulmonary oedema and ARDS is difficult both clinically and radiologically at the onset of the illness. But presence of prolonged severe hypoxia and persistent alveolar-interstitial shadows despite treatment with intravenous diuretics was more in favor of ARDS in our patient than pure pulmonary oedema. Management of myocarditis and acute heart failure follow standard guidelines with diuretics, angiotensin converting enzyme inhibitors and beta blockers. Place of steroid in acute myocarditis is debatable but it is commonly used by clinicians on empirical evidence and personal experience. European guideline on management of myocarditis recommends immunosuppression only in chronic virus negative myocarditis and inflammatory and autoimmune myocarditis [15]. None of the 6 reported patients with MSF and myocarditis had received steroids. However, we believe that steroids helped in treating our patient.

Oral doxycycline is the recommended antibiotics for rickettsial infections [13]. However, chloramphenicol which is a second line agent, is also widely used in many institutions in Sri Lanka. There are not many studies comparing the efficacy of chloramphenicol with other agents due to risk of major hematological adverse effects. In fact, CDC case report data suggest that patients with Rocky mountain spotted fever treated with chloramphenicol are at higher risk for death than persons who received a tetracycline [16]. In contrast to that our experience suggests that in severe spotted fever rickettsial infections, parenteral chloramphenicol can be used safely with good results like in this patient. Out of the 6 reported cases of MSF with myocarditis, chloramphenicol was included in treatment regimens of two patients [9]. Limitations of the our report includes the unavailability of histological evidence of myocarditis and not identifying the species due to lack of resources. Finally, this case highlights the need of prompt clinical diagnosis and treatment of spotted fever which can present with atypical features.

## Abbreviations

ARDS: Acute respiratory distress syndrome
MSF: Mediterranean spotted fever
NTproBNP: N-terminal pro b-type natriuretic peptide
PHH: Peripheral Hospital Hataraliyadda
THK: Teaching Hospital Kandy
